# Deep learning and hyperspectral features for seedling stage identification of barnyard grass in paddy field

**DOI:** 10.3389/fpls.2025.1507442

**Published:** 2025-02-07

**Authors:** Siqiao Tan, Qiang Xie, Wenshuai Zhu, Yangjun Deng, Lei Zhu, Xiaoqiao Yu, Zheming Yuan, Yuan Chen

**Affiliations:** ^1^ College of Information and Intelligent Science and Technology, Hunan Agricultural University, Changsha, Hunan, China; ^2^ College of Plant Protection, Hunan Agricultural University, Changsha, Hunan, China; ^3^ Hunan Engineering and Technology Research Centre for Agricultural Big Data Analysis and Decision-Making, Hunan Agricultural University, Changsha, Hunan, China; ^4^ Ecological Simulation Breeding and Phenotype ldentification Platform, Yuelu Mountain Laboratory of Hunan Province, Changsha, Hunan, China

**Keywords:** hyperspectral features, rice, barnyard grass, convolutional neural network, DeepBGS, sliding window

## Abstract

Barnyard grass, a pernicious weed thriving in rice fields, poses a significant challenge to agricultural productivity. Detection of barnyard grass before the four-leaf stage is critical for effective control measures. However, due to their striking visual similarity, separating them from rice seedlings at early growth stages is daunting using traditional visible light imaging models. To explore the feasibility of hyperspectral identification of barnyard grass and rice in the seedling stage, we have pioneered the DeepBGS hyperspectral feature parsing framework. This approach harnesses the power of deep convolutional networks to automate the extraction of pertinent information. Initially, a sliding window-based technique is employed to transform the one-dimensional spectral band sequence into a more interpretable two-dimensional matrix. Subsequently, a deep convolutional feature extraction module, ensembled with a bilayer LSTM module, is deployed to capture both global and local correlations inherent within hyperspectral bands. The efficacy of DeepBGS was underscored by its unparalleled performance in discriminating barnyard grass from rice during the critical 2-3 leaf stage, achieving a 98.18% accuracy rate. Notably, this surpasses the capabilities of other models that rely on amalgamations of machine learning algorithms and feature dimensionality reduction methods. By seamlessly integrating deep convolutional networks, DeepBGS independently extracts salient features, indicating that hyperspectral imaging technology can be used to effectively identify barnyard grass in the early stages, and pave the way for the development of advanced early detection systems.

## 1 Introduction

Barnyard grass, a pernicious weed with global ramifications, presents a formidable threat to agricultural ecosystems. Competing relentlessly with rice for essential resources such as light, water, nutrients, and space, it undermines the productivity of rice crops. Moreover, its presence fosters a conducive environment for pests and diseases, exacerbating the risk of diminished crop quality and yield. Early intervention is paramount in mitigating the spread of barnyard grass, ideally before it attains the 3-4 leaf stage, as its resistance to herbicides escalates with maturity. The eradication of mature barnyard grass poses considerable challenges, often necessitating excessive chemical interventions. Such practices not only foster herbicide resistance but also precipitate environmental degradation, jeopardizing the health of rice crops.

**Figure 1:**
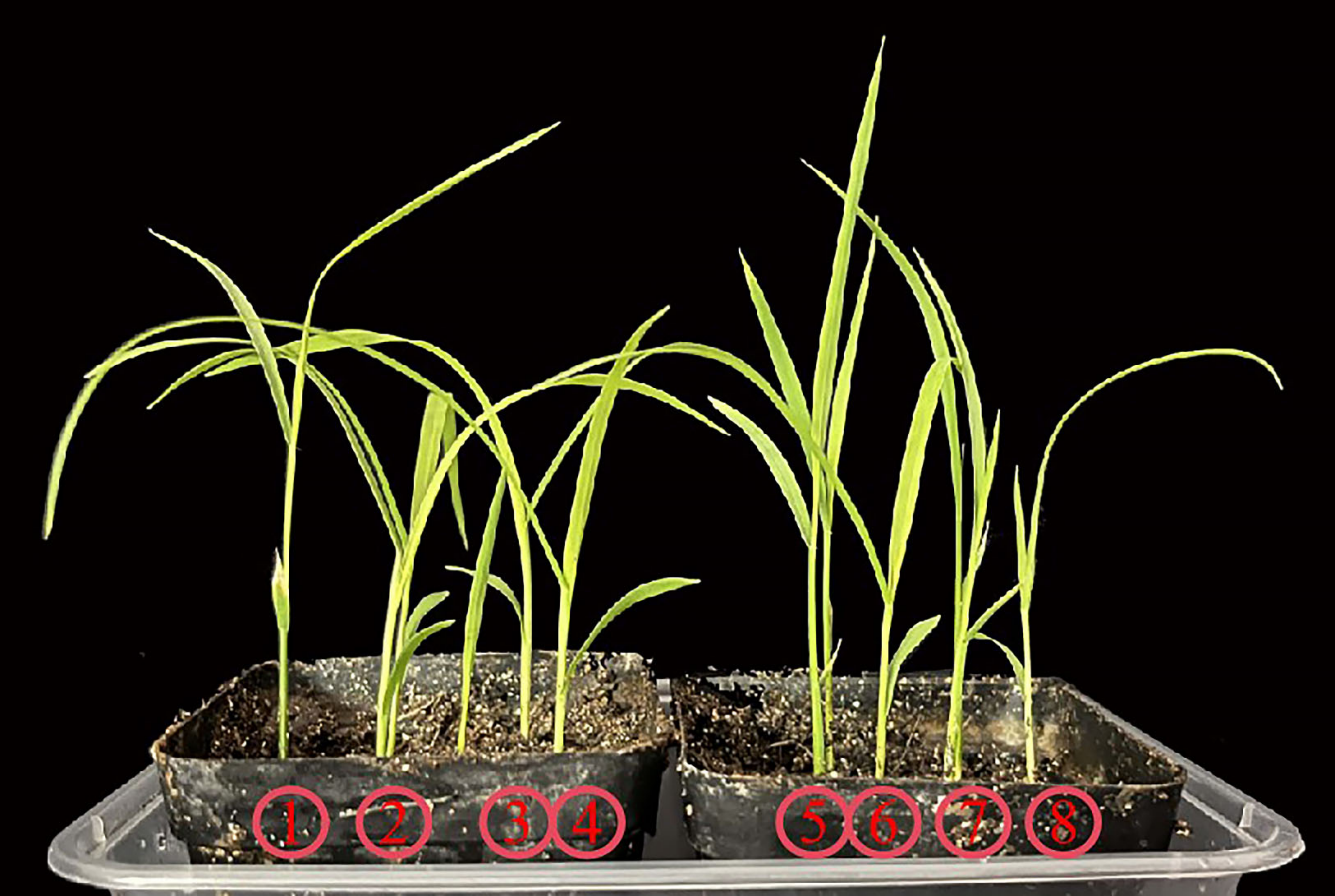
Comparison of seedling morphology between rice and barnyard grass. Plants designated as 1, 3, 5, and 7 represent rice, while those marked as 2, 4, 6, and 8 correspond to barnyard grass.

Precise field monitoring stands as the linchpin for timely barnyard grass control. However, conventional methods of manual identification are marred by their labor-intensive and time-consuming nature, rendering large-scale monitoring of barnyard grass outbreaks impractical. Hence, there arises an urgent need for the development of high-throughput and precise early detection techniques to effectively curb its proliferation, curtail herbicide usage, and enhance overall operational efficiency. In contrast to the conventional dryland weed identification methods, the recognition of weeds in rice fields predominantly relies on unmanned aerial vehicle (UAV) remote sensing image segmentation techniques. For instance, Huang et al. (2018) proposed a weed mapping and prescription map generation model, leveraging an enhanced fully convolutional network -4 (FCN-4) architecture for identifying Cyperus iric and Leptochloa chinensis in rice fields. Their approach yielded impressive overall accuracy and mean intersection over union (mean IU) scores of 0.9196 and 0.8473, respectively. Moreover, Ma et al. (2019) introduced a semantic segmentation method based on a fully convolutional network with the segmentation network (SegNet) model, achieving a mean average precision (MAP) of 0.927 by directly extracting features from initial RGB images and recognizing pixels corresponding to rice, background, and Sagittaria trifolia in paddy field images. Additionally, Lan et al. (2021) proposed improved feature fusion branch-bilateral segmentation network V2 (FFB-BiSeNetV2) models for real-time identification of rice weeds by UAV low-altitude remote sensing, achieving a pixel accuracy of 93.09% and a mean Intersection over union ratio of 80.28%. Furthermore, Kamath et al. (2020) investigated multiple classifier systems built using support vector machines (SVM) and random forest (RF) classifiers for classifying paddy crops and weeds from digital images, achieving an accuracy of 91.36%. Peng et al. (2022) devised the weed detection model based on RetinaNet, specifically addressing the challenge of overlapping between rice and various weed species. Their model achieved exceptional results with a high MAP of 94.1% on a dataset containing rice and eight distinct weed categories for object detection. Indeed, discerning barnyard grass from rice seedlings during their initial growth phases presents a formidable challenge, primarily owing to their remarkably similar visual appearances (**Figure 1**). This visual conundrum poses a significant obstacle for traditional visible-light imaging models, which often struggle to differentiate between the two with precision. Consequently, as of now, there exists no viable method leveraging visible light imaging models for accurately identifying barnyard grass seedlings.

Hyperspectral (HS) emerges as a potent tool for extracting both structural and physiological insights from plants, effectively circumventing the limitations of RGB imaging in distinguishing species with similar phenotypes (Mishra et al., 2017; Amigo and Grassi, 2019; Liu et al., 2021; Sarić et al., 2022). Li et al. (2021) demonstrated the efficacy of hyperspectral imaging data coupled with machine learning techniques in discriminating between two broadleaf weed species, Ranunculus acris (Giant buttercup) and Cirsium arvense (Californian thistle), achieving an impressive accuracy of 89.1%. Similarly, Diao et al. (2022) proposed a practical technical approach for rapidly training and identifying hyperspectral images of corn seedlings and weeds using a lightweight three-dimensional convolutional neural network, achieving an outstanding average recognition accuracy of 98.58%. It’s noteworthy that while Zhang et al. (2019) developed a SVM-based classification model leveraging six crucial spectral features selected by successive projections algorithm (SPA), yielding a commendable recognition rate of 97% for barnyard grass, weedy rice, and rice. However, their focus was primarily on the tillering stage rather than the seedling stage.

A plethora of wavebands in HS data offers rich information but also presents analytical challenges (Sarić et al., 2022). Crafting algorithms to dissect hyperspectral image data is pivotal in maximizing the potential of hyperspectral technology. In this study, we propose an automatic framework, deep learning-based identification model for barnyard grass in seeding stage (DeepBGS), for hyperspectral feature extraction based on convolutional neural networks (CNNs), aimed at precisely recognizing barnyard grass and rice at the seedling stage. Initially, preprocessed hyperspectral band sequences undergo segmentation into multiple overlapping subsequences, which are then transformed into a sequence matrix. Subsequently, a convolutional module featuring an attention mechanism is constructed to automatically extract correlation information between local and global bands. Independent tests unequivocally the superiority of DeepBGS over the reference model, achieving 98.18% accuracy in distinguishing barnyard grass and rice at the 2-3 leaf stage.

## 2 Materials and methods

### 2.1 Data acquisition

The study employed rice and barnyard grass specimens cultivated indoors under meticulously controlled conditions. These conditions ensured a temperature range of 25-28°C, humidity levels ranging between 40-70%, and a regulated 12-hour exposure to light. Only specimens of impeccable quality, devoid of any pest infestations or diseases, were chosen for leaf hyperspectral data collection. Spectral reflectance measurements of the leaves were conducted using a ground truth spectrometer (Field Spec Pro FR2500 spectrometer). This device boasted a wavelength range spanning from 350 to 2500 nm, with a sampling interval of 1.4 nm and a spectral resolution of 3 nm within 350-1000 nm range, and a sampling interval of 2.0 nm with a spectral resolution of 10 nm within 1000-2500 nm range. Leaf blade measurements were meticulously performed using leaf clamps within the canopy position of plants. Prior to each measurement, adjustments to the spectrometer parameters were meticulously carried out using the RS3 software, and spectral calibration against a whiteboard was conducted. To ensure robustness and accuracy, five consecutive samples were collected for each measurement, and the average value was calculated. A total of 660 samples were collected across three batches, comprising 313 rice and 347 barnyard grass specimens. The training set included 495 samples, incorporating data from the first two batches, with 243 rice and 252 barnyard grass samples. The test set, derived from the third batch, consisted of 70 rice and 95 barnyard grass specimens.

### 2.2 Data preprocessing

**Figure 2:**
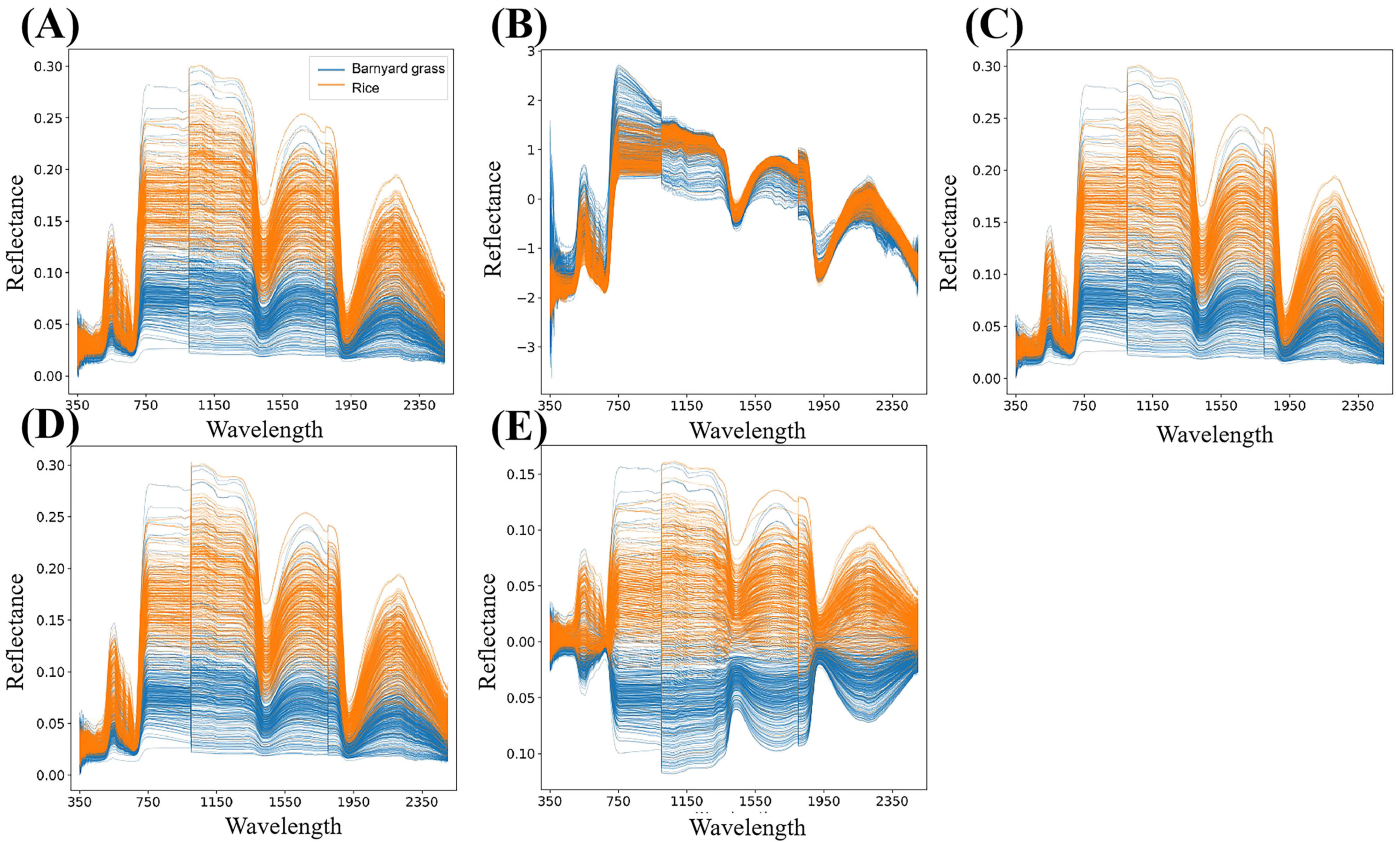
Raw spectral reflectance data and preprocessed data. The plots marked as **(A–E)** represent the reflectance map of the original spectral data, the SNV preprocessing result, the MA preprocessing result, the SG preprocessing result and the MC preprocessing result.

**Figure 3:**
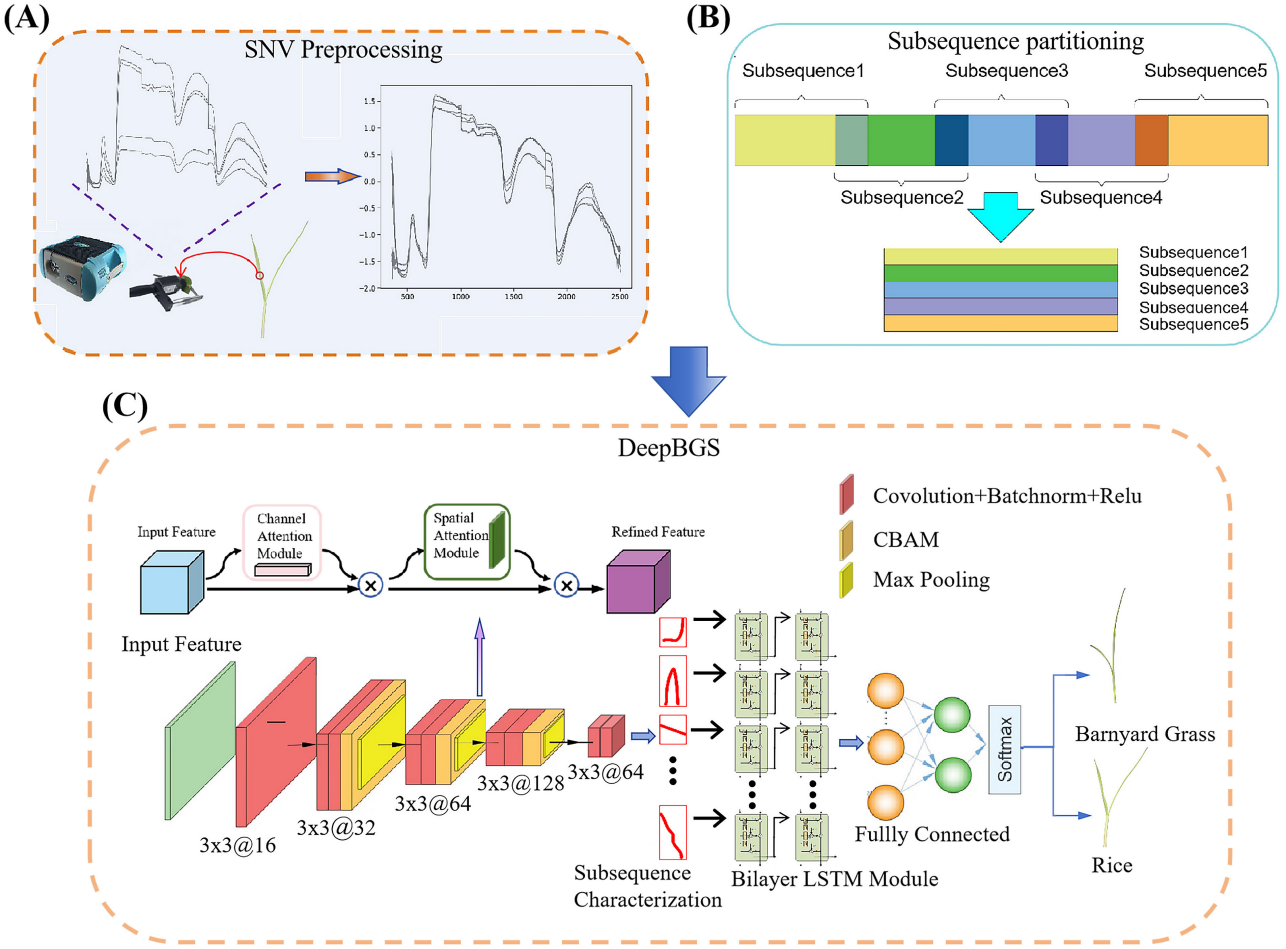
Workflow of DeepBGS. **(A)** illustrates the process of raw data acquisition and preprocessing. **(B)** shows the process of subsequence partitioning. **(C)** depicts the backbone of DeepBGS.

The dataset amassed in this investigation comprises spectral band reflectance data, with each sample encapsulating 2151 band reflectance values (**Figure 3A**). To fortify the reliability of the spectral data for subsequent analysis, four preprocessing methodologies were employed: standard normal variate transformation (SNV) (Bi et al., 2016), moving average (MA) (Timothy and Anuradha, 2023), savitzky golay smoothing (SG) (Luo et al., 2005), and mean centering (MC) (Ijomah, 2019). These methods served to mitigate data noise and rectify wavelength shifts, thus enhancing the overall quality and integrity of the spectral data. SNV, for instance, functions to rectify scale differences such as tilts or spikes, thereby accentuating the dynamic components of the spectral data. MA, on the other hand, operates by smoothing the dataset through point averaging within a window, effectively mitigating noise influence and suppressing periodic noise. SG, renowned for its efficacy in managing spikes and data variations, smooths the signal by attenuating high-frequency noise components. Lastly, MC plays a pivotal role in dataset rectification, eradicating biases stemming from measurement disparities, inherent variations, or substantial value discrepancies. The original spectral reflectance data and preprocessed data are shown in **Figure 2**.

### 2.3 Subsequence division

Consider a comprehensive breakdown of the preprocessing steps: employing a window length denoted as “*w*” and a sliding step designated as “*s*”, the preprocessed hyperspectral data, comprising “*m*” bands, is meticulously partitioned into (*m*-*w*)/*s*+1 segments. Notably, the final subsequence necessitates an extension in reverse to address any potential length discrepancies. Following this segmentation process, the divided subsequences undergo transformation into a two-dimensional matrix, as depicted in **Figure 3B**. This transformation sets the stage for the extraction of local band correlation features through the utilization of a sophisticated deep convolutional network.

### 2.4 Feature extraction module of DeepBGS

Following the subsequence transformation, each sample is epitomized by an *n*×*w* two-dimensional matrix, wherein *n* signifies the number of subsequences and *w* denotes the window length. This matrix stands as the foundational input layer of DeepBGS. Subsequently, the tensor of the *n*×*w* matrix traverses through the initial convolutional layer, endowed with rectified linear unit (ReLU) activation and a batch normalization (BN) layer. This layer, housing 16 filters of size 3×4 with a stride of 1, amplifies the dimensionality of the initial single-channel data, thus enhancing network expressiveness. The data then progresses through three consecutive feature extraction convolutional modules, each composed of two 3×3 convolutional layers, an attention mechanism layer, and a 2×2 max pooling layer. Notably, the attention mechanism layer harnesses a 3×3 convolutional kernel from the convolutional block attention module (CBAM), bolstering network feature expression and local region expression by integrating both channel and spatial attention. Meanwhile, the maximum pooling layer down samples the feature layer with a step size of 2×2. These three convolutional modules contain 32, 64, and 128 filters, respectively. Subsequent to this, the data is downscaled using two convolutional layers, each containing 64 filters, and then merged into a two-layer long-short term memory (LSTM) module with 32 hidden units, and two ReLU-based fully connected layers, featuring 32 and 64 output units, respectively, are incorporated. Regularization is implemented with a dropout probability of 50%, fortifying model generalization. Finally, the output layer employs the *Softmax* function to yield the probability of a sample belonging to rice. The parameters of DeepBGS are optimized using the *Adam Optimizer*, with a learning rate of 5*e*-6, a weight decay parameter of 0.0001, and training epochs and batch sizes set at 150 and 11, respectively. The structure of DeepBGS is shown in **Figure 3C**.

### 2.5 Reference models

Four traditional machine learning methods, including decision tree (DT) (Song and Lu, 2015), random forest (RF) (Breiman, 2001), SVM (Hearst et al., 1998) and extreme gradient boosting (XGBoost) (Osman et al., 2021) classifiers were used alongside four deep learning models—DeepBGS-NoLSTM, ResNet, VGG and multi-layer perceptrons (MLP) (Riedmiller, 1994) — to evaluate the performance of DeepBGS model. The DT model was implemented using the *R* package *part 4.1-15*, and the tree was pruned by the optimal *C* parameter value with the least cross-validated error. For the RF model, The *R* package *random Forest 4.6-14* was utilized, with parameters *ntree* and *mtry* set to 500 and $\sqrt{\text{No}.\text{ of features}}$ , respectively. The radial basis function (RBF) based-SVM model was executed using the Library for SVM, available at https://www.csie.ntu.edu.tw/%7Ecjlin/libsvm/index.html, A 10-fold cross-validation (10 CV) approach on the training set was used to optimize penalty parameters *C* (*C*∈[2^-5^, 2^15^]) and *g* parameter *g* (*g*∈[2^-15^, 2^3^]). XGBoost was implemented based on the Python *sklearn* library with default parameters. The MLP consists of three fully connected layers, where each hidden layer followed by a non-linear transformation employing the *ReLU* activation function. The output parameters of the initial two hidden layers were configured to have 1024 units, while the output layer employed the *softmax* activation function. DeepBGS-NoLSTM was akin to DeepBGS but the LSTM module was replaced by full connectivity layer. ResNet and VGG adopted 18-layer and 10-layer network architectures, respectively, which were implemented using the *PyTorch* framework.

### 2.6 Feature engineering

The primary challenge inherent in the analysis of multivariate hyperspectral data analysis lies in the necessity to represent a considerable number of wavebands, which significantly elevates the dimensionality of the data. Therefore, performing hyperspectral data dimension reduction becomes essential to mitigate data redundancy and extract valuable knowledge (Sarić et al., 2022). In this study, three types of feature engineering methods were explored, including feature dimensionality reduction, feature selection, and vegetation indices, to enhance the model’s prediction performance with high-dimensional bands.

Two techniques, principal component analysis (PCA) (Wold et al., 1987) and t-distributed stochastic neighbor embedding (t-SNE) (Belkina et al., 2019), were employed for feature dimensionality reduction. The determination of final dimensions was grounded on the principle of achieving optimal 10-fold cross-validated accuracy within the training set. Implementation of both PCA and t-SNE was facilitated through the Python *sklearn* library. Successive projections algorithm (SPA, https://gitee.com/aBugsLife/SPA) (Araújo et al., 2001) and consecutive adaptive reweighted sampling (Li et al., 2009) (CARS, https://gitee.com/aBugsLife/CARS) were utilized for the selection of important hyperspectral spectral bands. SPA employs a method that initially selects variables with the lowest covariance and redundancy while maximizing the projection vector in vector space. Subsequently, the final retained bands are determined based on the optimal principle of 10-fold cross-validated accuracy within the training set. Conversely, CARS utilizes a different approach. It began by retaining points with larger absolute weights of the regression coefficients in the partial least squares regression (PLS) model through adaptive reweighted sampling (ARS). It then iteratively builds PLS models based on the new subset, selecting wavelengths with the smallest root mean square error (RMSE) of cross-validation for the PLS model as the characteristic wavelengths after several calculations.

Ultimately, we diminished the dimensionality of the original data features by extracting spectral vegetation indices. A total of types of vegetation indices were extracted, including normalized difference vegetation index (NDVI), ratio vegetation index (RVI), triangular vegetation index (TVI), photochemical reflectance index (PRI), and normalized pigment chlorophyll ratio index (NPCI). These indices were constructed using the following formula:


(1) $$\text{NDVI}\left({\text{r}}_{1},{\text{r}}_{2}\right)=\frac{\text{NIR}({\text{r}}_{1})-\text{VR}({\text{r}}_{2})}{\text{NIR}({\text{r}}_{1})+\text{VR}({\text{r}}_{2})}$$

(2) $$\text{RVI}\left({\text{r}}_{1},{\text{r}}_{2}\right)=\frac{\text{NIR}({\text{r}}_{1})}{\text{VR}({\text{r}}_{2})}$$

(3) $$\text{TVI}=0.5\times \left[120\times \left({\text{R}}_{750}-{\text{R}}_{550}\right)-200\times \left({\text{R}}_{650}-{\text{R}}_{550}\right)\right]$$

(4) $$\text{PRI}=\frac{{\text{R}}_{531}-{\text{R}}_{570}}{{\text{R}}_{531}+{\text{R}}_{570}}$$

(5) $$\text{NPCI}=\frac{{\text{R}}_{680}-{\text{R}}_{430}}{{\text{R}}_{680}+{\text{R}}_{430}}$$

The bands *r*_1_ and *r*_2_ correspond to distinct regions of the light spectrum, with *r*_1_ encompassing the near-infrared region and *r*_2_ encompassing the infrared region. Specifically, *r*_1_ comprises wave lengths of 724nm, 738nm, 750nm, 764nm, 776nm, 790nm, 802nm, and 814nm, while *r_2_* consists of 601nm, 605nm, 614nm, 627nm, 6336nm, 644nm, 652nm, 660nm, 669nm, and 677nm. Leveraging these bands, we derived 80 features for the NDVI and 80 features for the RVI. Additionally, we formulated a TVI feature utilizing wavelengths 750nm, 550nm, and 650nm, a PRI feature using wave-lengths 531nm and 570nm, and an NPCI feature using wavelengths 680nm and 430nm. In total, 163 vegetation index features were extracted from the hyperspectral data.

### 2.7 Evaluation indicators

Accuracy (ACC), area under the curve (AUC), and matthews correlation coefficient (MCC) are utilized as evaluation metrics to assess the performance of the model predictions. These metrics are defined as follows:


(6) $$\text{ACC}=\frac{\text{TP}+\text{TN}}{\text{TP}+\text{TN}+\text{FP}+\text{FN}}$$

(7) $$\text{MCC}=\frac{\left(\text{TP}+\text{TN}\right)-\left(\text{FN}+\text{FP}\right)}{\sqrt{\left(\text{TP}+\text{FN}\right)\times \left(\text{TN}+\text{FP}\right)\times \left(\text{TP}+\text{FP}\right)\times \left(\text{TN}+\text{FN}\right)\;}}$$

Where TP, TN, FP, and FN represent true positive, true negative, false positive, and false negative, respectively. The receiver operating characteristic (ROC) curve illustrates the true-positive rate against the false-positive rate (1 - specificity) for various thresholds. The AUC of the ROC curve is analyzed to provide a comprehensive metric for evaluating prediction methods. Higher values of ACC, MCC, and AUC indicate better prediction ability.

## 3 Results

### 3.1 A conventional machine learning-based model for classifying barnyard grass and rice at seedling stage

**Figure 4:**
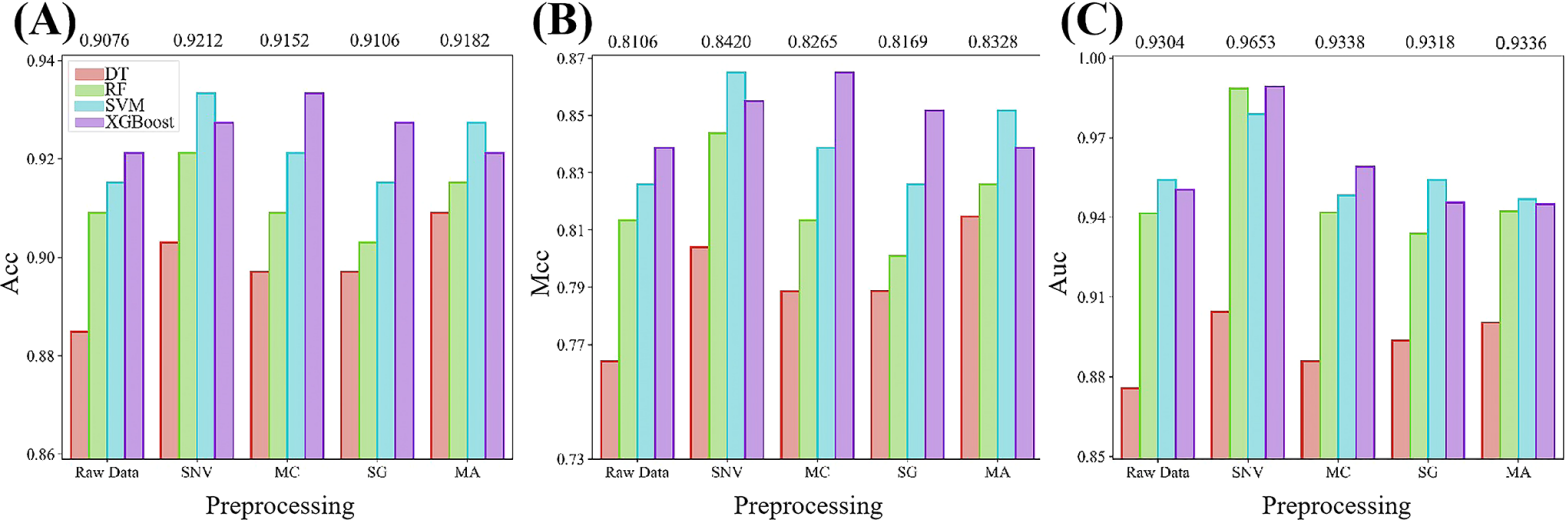
Comparative analysis of various evaluation metrics for traditional machine learning with different preprocessing techniques. **(A–C)** show the performance comparison of ACC, MCC, and AUC respectively. The numbers above the bars in the figure indicate the average scores of the metrics for the five models under each preprocessing algorithm.

We initially assessed the performance of traditional machine learning models in aiding the hyper-spectral differentiation between barnyard grass and rice during the seedling stage. Additionally, we investigated the impact of various data preprocessing methods on model performance. Hyperspectral data, subjected to diverse various preprocessing techniques, constituted the input data for the traditional machine learning models, and the prediction results for independent test set of each model are illustrated in **Figure 4**. Among the four traditional machine learning models, XGBoost exhibited the most predictive performance, with average ACC, MCC, and AUC values reaching 0.9261, 0.8499, and 0.9579, respectively. Following XGBoost, the SVM model emerged as the second best, achieving average ACC, MCC, and AUC of 0.9224, 0.8415, and 0.9565, respectively. In contrast, the DT model displayed the lowest prediction accuracy, with average ACC, MCC, and AUC metrics of only 0.8982, 0.7920, and 0.8920, respectively. Compared to the original data, all four types of preprocessing methods effectively improved the model prediction performance, with SNV exhibiting the most notable improvement. SNV yielded average ACC, MCC, and AUC values of 0.9212, 0.8420, and 0.9653, respectively. Overall, irrespective of preprocessing or the choice of machine learning algorithms, the ACC and MCC of the hyperspectral-based machine learning model consistently surpassed 0.88 and 0.76, respectively, in distinguishing between barnyard grass and rice during the seedling stage. These findings underscore the potential of hyperspectral data in distinguishing between barnyard grass and rice at the seedling stage.

### 3.2 Classification model based on feature dimensionality reduction

**Figure 5:**
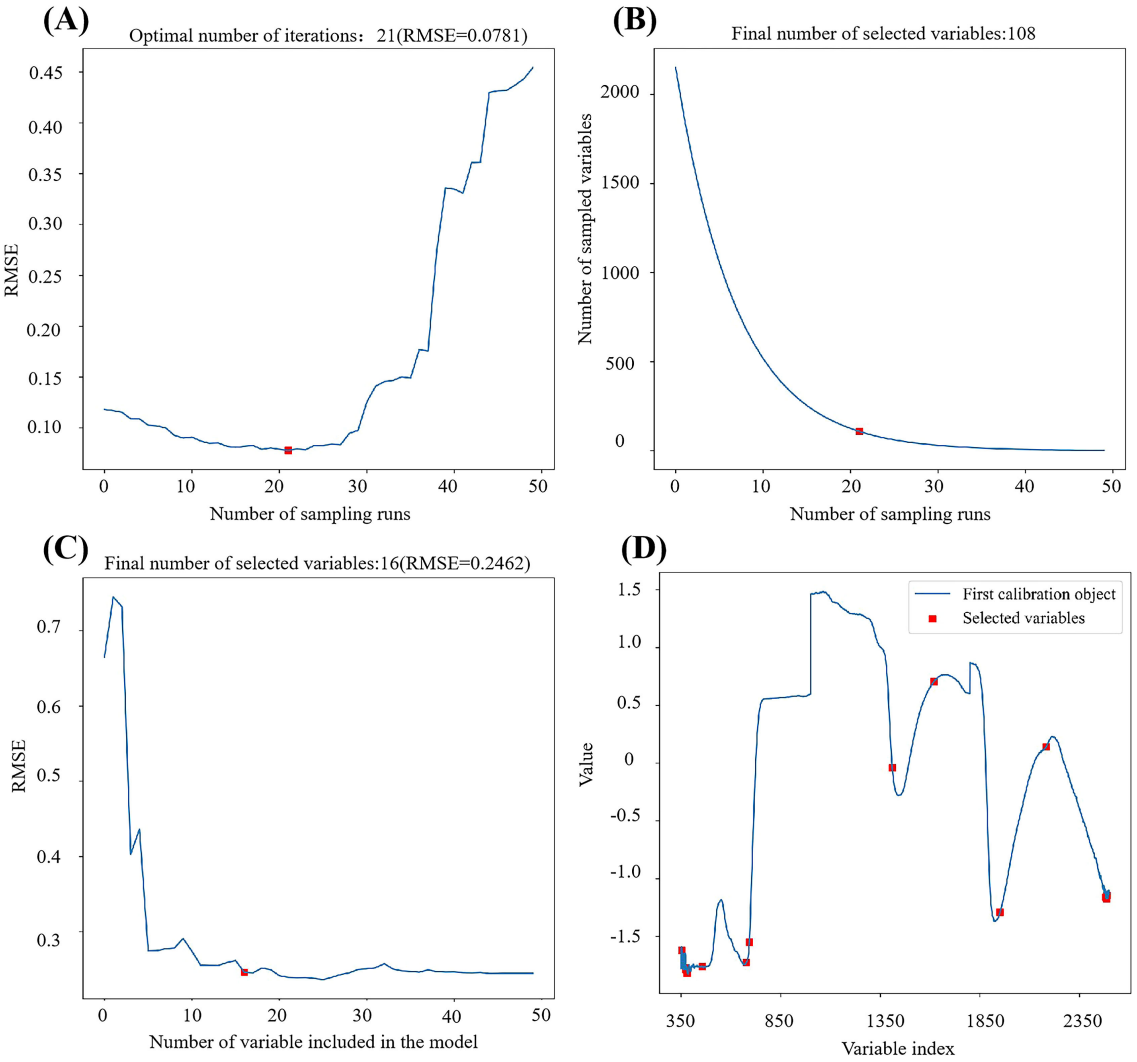
Feature selection process. The plots marked as **(A, B)** represent the CARS feature selection process, and those marked as **(C, D)** represent the SPA feature selection process. **(A)** shows how the number of selected variables changes as the sample size increases and **(B)** illustrates the variation in cross-validation RMSE values with an increasing number of samples. the process of variable selection and the results of variable selection based on SPA algorithm are presented in the diagrams labeled **(C, D)**, respectively.

To enhance the predictive accuracy of our models, we employed three distinct feature engineering strategies aimed at reducing its dimensionality: feature dimensionality reduction, feature selection, and vegetation index conversion. We applied spectral data after SNV pre-processing for both feature dimensionality reduction and feature selection, while vegetation index conversion was conducted based on the original spectral reflectance data. For PCA and t-SNE, we determined the optimal retained dimensionality by evaluating 10-fold cross-validation ACC using XGBoost on training set. PCA retained thirty principal components, while t-SNE retained twelve features. The SPA band selection process and the CARS band selection process were visualized in **Figure 5**. As the number of characteristic bands increases, the RMSE value decreases and levels off. When the RMSE stabilizes and reaches its optimum, the value is 0.2462. At this point, the SPA retained 16 bands, comprising wavelengths such as 678nm, 457nm, 693nm, 1410nm, 2182nm, 1951nm, 354nm, 2481nm, 376nm, 2483nm, 381nm, 374nm, 1619nm, 2485nm,2486 and 2488nm. In **Figure 5A**, the number of bands gradually decreases and stabilizes, while **Figure 5B** shows a decreasing trend followed by an increase. At a sampling number of 21, the RMSECV reaches its minimum, leading to the selection of the bands obtained from the 21^th^ sampling as the characteristic wavelengths, totaling 108 wavelengths. Additionally, we derived 163 vegetation index features through band conversion.

Drawing from important features retained by various feature engineering strategies, we constructed traditional machine learning models and presented the results of independent tests in **Table 1**. Utilizing vegetation index features, the average ACC and MCC of the four machine learning models were 0.9152 and 0.8311, respectively, notably lower than those of the prediction model based on the full band, where the average ACC and MCC were 0.9212 and 0.8420, respectively. Following PCA downscaling, the model prediction accuracy showed slight improvement Compared to the full band except for the DT model, registering an average ACC and MCC of 0.9353 and 0.8712, yet, the DT model experiences a slight reduction, primarily observed in the decline of ACC and MCC to 0.8242 and 0.6583. However, t-SNE, SPA, and CARS feature downscaling methods exhibited enhanced model prediction performance compared to full-band modeling. Among these, the t-SNE feature dimensionality reduction method demonstrated the most significant improvement, with an average ACC and MCC of 0.9409 and 0.8800, respectively. The SVM model based on the retained features of SPA exhibited the best prediction outcomes, attaining an ACC and MCC of 0.9455 and 0.8886, respectively. These findings indicate the effectiveness of feature dimensionality reduction methods, particularly t-SNE, in enhancing the performance of traditional machine learning models.

**Table 1:** Performance impact of different feature dimensionality reduction methods on traditional machine learning models.

Feature engineering strategies	Evaluation Measures	Dt	RF	SVM	Xgboost	Average
PCA	ACC	0.8242	0.9394	0.9333	0.9333	0.9076
	MCC	0.6583	0.8806	0.8639	0.8691	0.8180
T-SNE	ACC	0.9455	0.9394	0.9394	0.9394	0.9409
	MCC	0.8896	0.8779	0.8767	0.8759	0.8800
SPA	ACC	0.9030	0.9091	0.9455	0.9333	0.9227
	MCC	0.8112	0.822	0.8886	0.8664	0.8471
CARS	ACC	0.9394	0.9333	0.9273	0.9394	0.9349
	MCC	0.8779	0.8639	0.8519	0.8759	0.8674
Vegetation indices	ACC	0.9152	0.8909	0.9212	0.9333	0.9152
	MCC	0.8329	0.7867	0.8385	0.8664	0.8311

### 3.3 DeepBGS-based model for classification of barnyard grass and rice at seedling stage

**Figure 6:**
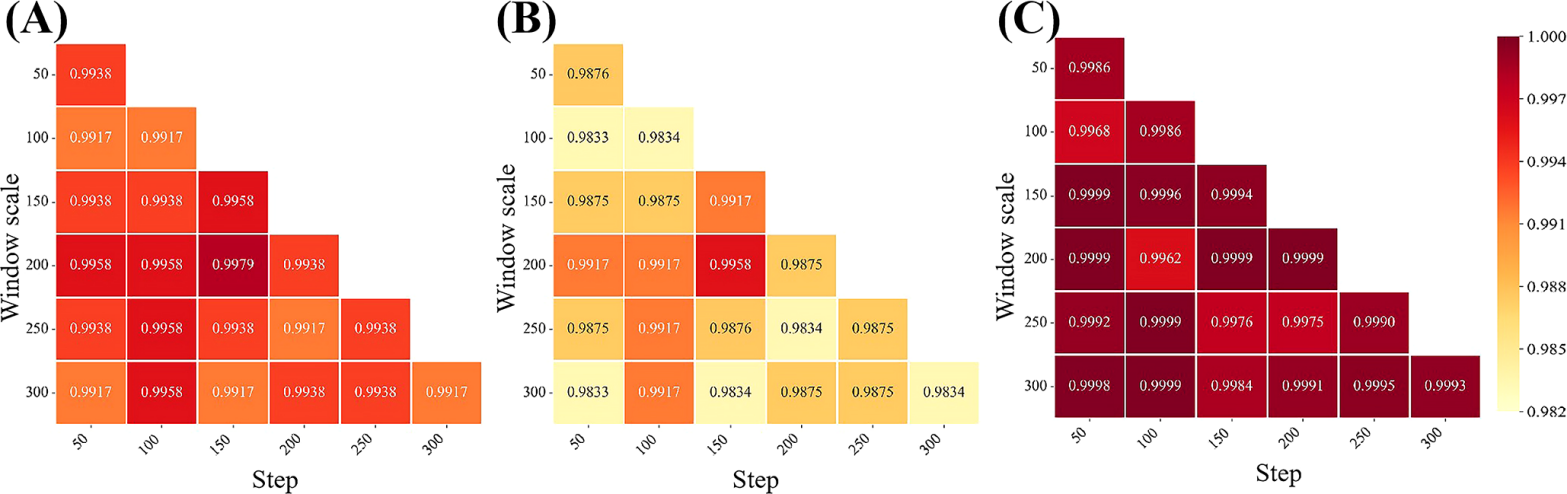
Effects of various combinations of window length and sliding step size on model performance. **(A–C)** show the performance of ACC, MCC, and AUC respectively. The horizontal axis is the step size, and the vertical axis is the window size.

Transforming linear hyperspectral reflectance data into 2D matrix data through subsequence division is crucial for extracting spectral global and local correlation features by DeepBGS. Therefore, our initial focus lies in optimizing the subsequence window length and sliding step length to select appropriate parameters for the DeepBGS model. Through grid optimization grounded in 10-fold cross-validation of the training set, we explored various combinations of window lengths and sliding step lengths within the range of 50-300, with a step length of 50. Previous results have shown that the models achieve the best prediction performance following the preprocessing of original data using SNV. Hence, DeepBGS modeling exclusively relies on SNV preprocessed data, and the 10-fold cross-segmentation of the training set remains consistent across all combinations of window length and sliding step length. The model prediction outcomes across different combinations of window length and sliding step length were visualized in **Figure 6**. Notably, when the window length is set to 200 and the sliding step length to 150, the DeepBGS model attained its highest training performance, with ACC, MCC, and AUC reaching 0.9979, 0.9958, and 0.9999, respectively.

**Figure 7:**
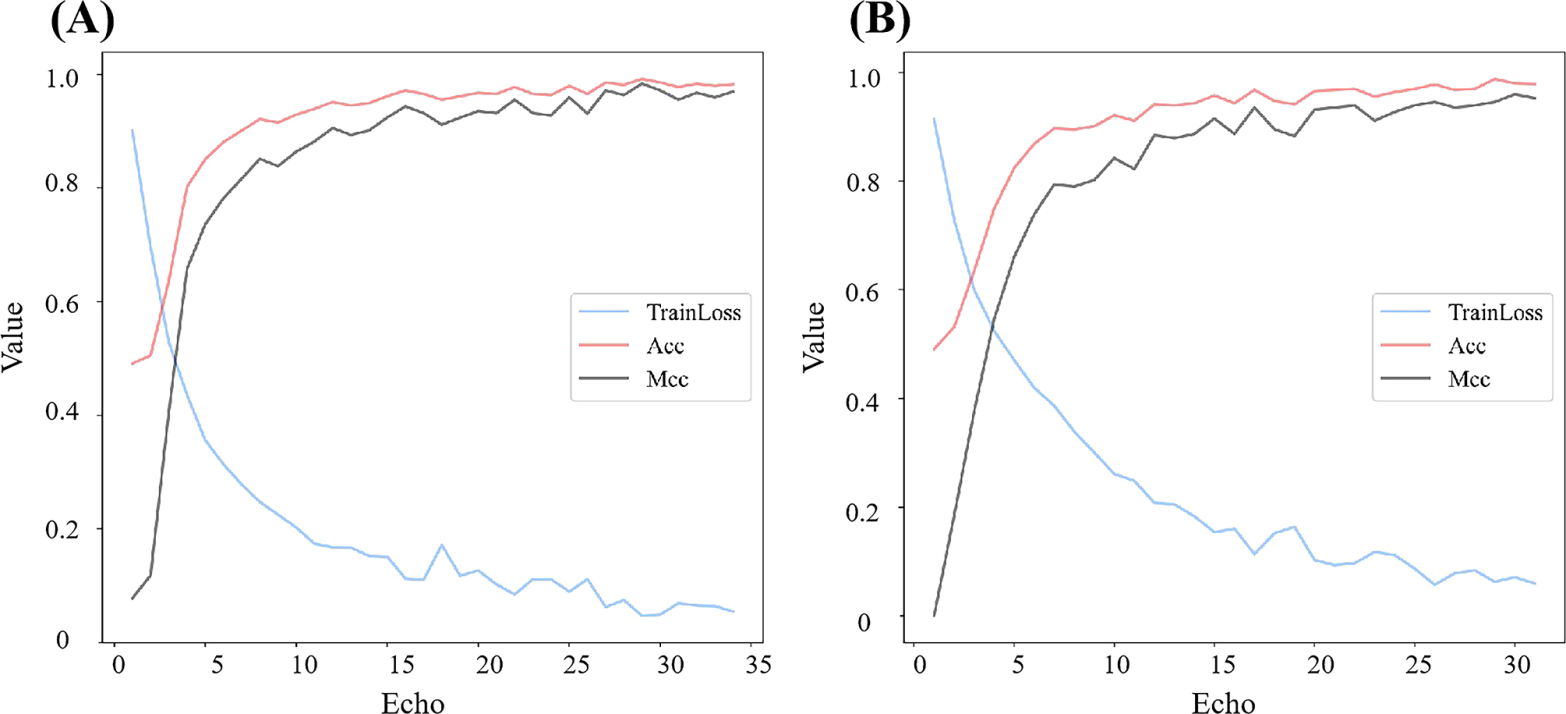
Training performance of Resnet models with different depths. **(A)** is Resnet18 and **(B)** is Resnet50.

Based on the optimal combination of window length and sliding step (*w* = 200, *s* = 150), we further compared the performance of deep convolutional networks for extracting sequence matrix features. As illustrated in **Table 2**, **Figure 7**, all five deep learning models demonstrate robust capabilities in distinguishing seedling barnyard grass from rice, with ACC surpassing 0.93 and MCC exceeding 0.87. Remarkably, the DeepBGS model emerges as the top performer, achieving a flawless differentiation rate of 98.18%. All 95 barnyard grass samples were correctly classified, with only 3 out of 70 rice samples misclassified as barnyard grass (**Table 3**). In contrast, the DeepBGS-NoLSTM model, which excludes the LSTM module, experiences a slight reduction in independent test accuracy, primarily observed in the decline of ACC and MCC to 0.9697 and 0.9379. The VGG model, without the batch normalization layer, exhibited a more pronounced decrease in prediction performance, with ACC and MCC plummeting to only 0.9515 and 0.9014, respectively, comparable to traditional machine learning models. And the MLP module attained ACC and MCC scores of 0.9394 and 0.8779. Meanwhile, the Resnet18 model, which featured an 18-layer network, attained ACC and MCC scores of 0.9636 and 0.9256, respectively, underscoring its efficacy in distinguishing between barnyard grass and rice at the seedling stage. However, augmenting the network depth to 50 layers resulted in diminished prediction accuracy for the Resnet50 model, with ACC and MCC declining to 0.9455 and 0.8886, respectively.

**Table 2:** Independent test performance of deep learning models.

Evaluation Measures	DeepBGS	DeepBGS-NoLSTM	VGG	Resnet18	Resnet50	MLP
ACC	0.9818	0.9697	0.9515	0.9636	0.9455	0.9394
MCC	0.9632	0.9379	0.9014	0.9256	0.8886	0.8779

**Table 3:** Confusion matrix for the prediction results of the DeepBGS.

		Predicted classes	
		Barnyard grass	Rice
Actual classes	Barnyard grass	95	0
	Rice	3	67

## 4 Discussion

Controlling barnyard grass is particularly challenging due to its strong resistance to herbicides. Effective control of barnyard grass in the early stages can significantly reduce both economic and environmental costs. Establishing a dependable early identification system for weeds is paramount, with HS emerging as a promising avenue over RGB imaging. Unlike the latter, HS focuses on phytochemical composition indicators rather than solely shape, size, and color, offering distinct advantages (Farooq et al., 2018). Hyperspectral data furnishes intricate reflectance information across numerous narrow spectral bands. However, grappling with the high redundancy and multicollinearity inherent in these bands presents a formidable obstacle. Extracting actionable insights from such data stands as a pivotal requirement for effective weed early identification. Prior studies, exemplified Zhang et al. (2019), have demonstrated promising outcomes utilizing methodologies like the SPA in tandem with weighted SVM models for the identification of barnyard grass, weedy rice, and rice, achieving high recognition rates. In the present study, we employed three feature engineering methods: feature dimensionality reduction, feature selection, and vegetation index conversion. While certain methods like PCA and vegetation index conversion failed to bolster model accuracy, others such as t-SNE for dimensionality reduction and feature selection techniques like CARS and SPA exhibited enhancements in prediction performance. However, achieving accurate differentiation between barnyard grass and rice at the seedling stage remains an ongoing challenge.

This study compares the performance of traditional machine learning models and deep learning methods in identifying barnyard grass and rice at the seedling stage. Unlike traditional approaches, deep learning eliminates the need for complex feature engineering, autonomously extracting meaningful patterns from raw data to achieve superior classification results (Perez-Sanz et al., 2017; Sarić et al., 2022). Although a defining feature of deep learning is that the input data can be unstructured (Murphy et al., 2024), the non-imaging hyperspectral data in this study is inherently structured. When a simple fully connected network is used, its prediction accuracy reaches 0.9394, comparable to that of traditional machine learning models. To fully capture both local and global associations among hyperspectral bands, we introduce a subsequence conversion method based on sliding windows. This approach transforms the hyperspectral bands from one-dimensional vectors into two-dimensional matrices, enabling them to serve as input for a deep learning model that combines convolutional networks and LSTM. This method leverages the robust feature extraction capabilities of deep learning. Building on the ResNet model, we examined the effect of network depth on model performance and found that excessive depth can lead to overfitting. As shown in **Figure 7**, the Resnet18 model, despite its lower network depth, achieved stable training results after only 31 rounds of training, with both training ACC and MCC stabilizing at 0.9778 and 0.9556, respectively. In the independent test, the ACC and MCC remained stable at 0.9636 and 0.9256, respectively. Conversely, after 28 rounds of training for the Resnet50 model with increased network depth, although the loss stabilized, the training ACC and MCC reach slightly lower values of 0.9687 and 0.9394, respectively. However, the final independent test performance exhibited more significantly decline, with the ACC and MCC dropping to 0.9455 and 0.8886, respectively. This phenomenon may arise from the limited training data, which makes models with complex network architectures more susceptible to overfitting. Consequently, DeepBGS in this study employs only a 9-layer convolutional module.

This study investigates the hyperspectral characteristics distinction between barnyard grass and rice at the seedling stage and proposes a deep learning-based framework for hyperspectral feature extraction. However, the current approach relies solely on indoor non-imaging methods to extract spectral features, and the limited number of training samples necessitates further work before it can be applied in the field. First, the spectral data from crop canopies in the field are filtered by various factors, such as sunlight, angle, cloud absorption, and shadows, leading to significant differences from the controlled laboratory environment. Therefore, acquiring more field-labeled samples is crucial for enhancing the model’s practical applicability. Additionally, transfer learning may offer a solution for developing effective models with limited training data. For real-time weed recognition models deployed in the field, those with lower costs and higher computational efficiency may be preferable, even at the expense of reduced accuracy (Murphy et al., 2024). Therefore, extending the model to field-based hyperspectral imaging data and using tools such as deep learning important features (DeepLiFT) and class activation mapping (CAM) to enhance the interpretability of deep learning models—by identifying key spectral bands—could guide the development of more cost-effective multispectral devices, significantly advancing early weed detection technology in the field.

## 5 Conclusion

Barnyard grass resistance to pesticides increases with growth, making early identification and precise control essential for reducing pesticide use and advancing precision agriculture. To address the challenge of distinguishing barnyard grass from rice seedlings at the early growth stage, this study explores hyperspectral imaging for identification. A combined model incorporating multiple spectral preprocessing techniques, feature engineering methods, and traditional machine learning was evaluated, achieving a maximum classification accuracy of 93.94%. Finally, we developed a deep learning-based framework for extracting hyperspectral features, achieving 98.18% accuracy in distinguishing seedling barnyard grass from rice.

## Data Availability

The datasets presented in this study can be found in online repositories. The names of the repository/repositories and accession number(s) can be found below: Data will be made available at https://github.com/dejavu1021/DeepBGS.
